# Efficacy and Safety of Peripherally Acting μ-Opioid Receptor Antagonist (PAMORAs) for the Management of Patients With Opioid-Induced Constipation: A Systematic Review

**DOI:** 10.7759/cureus.16201

**Published:** 2021-07-05

**Authors:** Martina Rekatsina, Antonella Paladini, Asbjørn M Drewes, Farrah Ayob, Omar Viswanath, Ivan Urits, Oscar Corli, Joseph Pergolizzi Jr, Giustino Varrassi

**Affiliations:** 1 Pain Management, Whipps Cross Hospital Barts Health NHS, London, GBR; 2 Department of Clinical Medicine, Public Health and Life Science (MESVA), University of L'Aquila, L'Aquila, ITA; 3 Department of Gastroenterology and Hepatology, Aalborg University Hospital, Aalborg, DNK; 4 Anesthesia, Barts Health, London, GBR; 5 Pain Management, Valley Pain Consultants - Envision Physician Services, Phoenix, USA; 6 Department of Anesthesia, Critical Care and Pain Medicine, Beth Israel Deaconess Medical Center, Harvard Medical School, Boston, USA; 7 Pain and Palliative Care Research Unit, Mario Negri Institute IRCCS, Milano, ITA; 8 Research Department, Nema Research, Inc., Naples, USA; 9 Research, Paolo Procacci Foundation, Roma, ITA

**Keywords:** opioids, pain, opioid induced constipation, opioid-induced bowel dysfunction, pamora, peripherally acting m-opioid receptor antagonist

## Abstract

In treating chronic and acute pain, opioids are widely used. Although they do provide analgesia, their usage does come with adverse events (AEs). One of the most burdensome is opioid-induced bowel dysfunction, and more specifically opioid-induced constipation (OIC). The pathogenesis of these AEs is well known as the consequence of the action of opioids on m-receptors in the enteric nervous system. In recent years, medicines counteracting this specific action at the receptors have been registered for clinical use: the peripherally acting μ-opioid receptor antagonists (PAMORAs). The knowledge of their comparative efficacy and tolerability is very important for physicians and patients in opioid therapy. This systematic review of the existing literature on PAMORAs aimed to study the relative clinical advantages and disadvantages.

The most important data banks, including “PubMed,” “Embase,” “CT.gov,” “ICTRP” and “CINAHL” were used to find the published material on PAMORAs. The selected publications were examined to systematically analyze the efficacy and safety of the four existing PAMORAs.

All of the medications are superior to placebo in reducing OIC. There are few published data on alvimopan used to treat OIC, and it is only indicated for the treatment of post-abdominal surgery ileus. Methylnaltrexone is studied mainly in its subcutaneous (SC) formulation. When used in its oral formulation, it seems more rapid than naloxegol and placebo in the reduction of OIC. Naldemedine is able to produce more spontaneous bowel movements (SBMs) when compared to alvimopan and naloxegol.

Tolerability was found to be similar for all of them. In particular, they affect the gastrointestinal tract (GI), with flatulence and diarrhea, especially at high dosages. For some of them, nasopharyngitis and abdominal pain were observed as treatment adverse effects (TEAs). Several cardiovascular TEAs were reported after methylnaltrexone use, but it is not clear whether they were consequences of the drug or related to the general conditions of the patients.

Considering the existing data, naloxegol and naldemedine seem to be the best choices, with a higher number of spontaneous bowel movements following naldemedine administration.

## Introduction and background

Opioids are powerful analgesics that have been used for centuries in the treatment of acute and chronic cancer and non-cancer pain [1]. Among their common side effects, the most bothersome and debilitating are those associated with opioid-induced bowel dysfunction (OIBD). This includes opioid-induced constipation (OIC) [2,3], defined as a change in baseline bowel habit or defecatory patterns following initiation, modification, or increase of opioid therapy [1]. OIC is a common side effect, yet under-recognized and under-treated [2]. This ongoing burden emphasizes the need to identify more efficacious constipation therapies for the chronic pain patient population treated with opioids [4] as effective pharmacologic therapy for OIC is considered an unmet need [3].

Pathogenesis of OIC

The pathogenesis of OIBD and OIC is attributed to the action of opioids on their receptors in the gastrointestinal tract [1]. Opioid receptors (μ, κ, and δ) are spread throughout the gastrointestinal tract (GI) from the mid-esophagus to the rectum and are involved in a variety of cellular functions [5]. In humans, μ-receptors are thought to be of utmost importance for the homeostatic functions of the enteric nervous system [6]. Endogenous ligands play a role in the normal regulation of GI function, but opioid receptors are also activated by exogenous opioids [6,7]. Opioid agonists administration results in modifications of the normal GI physiology, with segmentation, increased tone, and uncoordinated motility reflected in constipation. Also, opioids’ administration results in increased absorption and decreased secretion of fluids in the gut, leading to dry feces and less propulsive motility [8]. They increase sphincter tone, which may cause symptoms such as sphincter of Oddi spasms and hampered rectal evacuation [8-10]. Opioid antagonists counteract the effects of opioids in the human gut on motility, fluid transport, and sphincter function [8].

Prevalence of OIC

OIC is the most common subtype of OIBD that occurs in 51-87% of patients receiving opioids for cancer and between 41% and 57% of patients receiving opioids for chronic noncancer pain [11-13]. A recent "real-world" multicenter, observational study assessed cancer patients on opioids for the prevalence of OIC [14]. The authors utilized some different diagnostic criteria for OIC. They concluded that 59% of patients had clinical OIC, 2.5% had another cause of constipation, and 19% did not have constipation but were assuming laxatives [14]. A multicenter cross-sectional observational study showed that approximately two out of three patients with chronic opioid intake experienced a degree of constipation that was problematic for the patient, while more than four out of five patients were considered constipated according to the physician's subjective assessment despite laxative use [15].

Effects of opioids

Dose, frequency, and duration of opioid therapy influence the likelihood of OIC symptoms [16]. Bell et al. [17] mentioned that daily use of opioids resulted in constipation in 81% of patients, whereas patients using opioids two to three times per week reported constipation in less than 50% of the cases. Moreover, the route of opioid intake seems to play a role, as transdermal preparations of fentanyl and buprenorphine may be associated with a lower incidence of OIC than oral opioids both in cancer and non-cancer patients [18]. Results should, however, be interpreted cautiously as these studies included small patient materials and had several inherent flaws, and no matter what the administration route is the opioids will reach the gut. In addition, OIC does not spontaneously decrease over time due to tolerance (for the colon only) but persists with unchanged prevalence [19].

OIC was not associated with demographic factors, cancer diagnosis, performance status, or opioid dosage. However, it was associated with specific opioid analgesics, namely tramadol, tapentadol, and transdermal buprenorphine which both led to less constipation [14]. The study confirms that OIC is common among patients with cancer pain and is associated with a spectrum of physical symptoms, a range of psychological symptoms, and an overall deterioration in the quality of life [14].

Impact

OIC could lead to pain exacerbation, longer hospitalization, frequent changes in opioids [3,15] and laxative treatment [15,20], higher healthcare resource utilization, and other extra costs [20,21]. Moreover, OIC has a negative impact on work productivity (as reflected by missed days and impairment while working) as well as health-related quality of life [3,14,15,17].

Diagnosis

To identify OIC, the Bowel Function Index (BFI), a physician-administered, easy-to-use scale can be utilized to objectively identify patients who need more aggressive treatment [22,23]. For research studies, patients meeting the criteria for OIC should not be given a diagnosis of functional constipation (FC) because it is difficult to distinguish between opioid side effects and other causes of constipation. However, clinicians recognize that these two conditions may overlap [24]. The Rome IV diagnostic criteria for opioid-induced constipation is an updated, systematized definition of OIC and is also a very useful tool (Table 1) [23]. According to a recent observational study, the Rome IV diagnostic criteria had an accuracy of 81.9%, which is extremely high [14].

**Table 1:** Diagnostic criteria. Roma IV diagnostic criteria [23].

1. New, or escalating, symptoms of constipation when initiating, changing, or increasing opioid therapy that must include two or more of the following:
a. Straining during more than one-quarter of defecations
b. Lumpy or hard stools (BSFS 1–2) more than one-quarter of the time.
c. Sensation of incomplete evacuation more than one-quarter of the time.
d. Sensation of anorectal blockage/obstruction in more than one-quarter of defecations.
e. Manual maneuvers to facilitate more than one-quarter of defecations.
f. Fewer than three spontaneous bowel movements per week.
2. Loose stools rarely present without the use of laxatives.

Management

After identification of OIC, it is important to assess the patient clinically, identify the reason for an opioid prescription, the current doses as well as differentiate OIC from pre-existing constipation exacerbated by the opioids [1]. Addressing exacerbating factors, including concurrent constipating medications (calcium channel blockers, diuretics, etc.) is also crucial [1]. Some general management could include lifestyle modification (increase fluid intake, exercise) and addition of standard laxatives (osmotic agents and stimulants) although never documented to be beneficial in controlled studies. Change to a different opioid or change route of administration would be a different option, but again the evidence is sparse [18].

Educational strategies need to be developed to improve the knowledge base of healthcare providers on the identification [1] and challenging management of OIC [3]. However, despite early management, constipation might still develop and persist [4].

Should the above be the case, opioid-receptor antagonists can alleviate the adverse effects of opioids on GI functions [25]. Some agents that cross the blood-brain barrier, like naloxone, antagonize the central analgesic effects [25]. Other medicines not crossing this barrier (or are actively transported out of the central nervous system) block only peripheral opioid receptors, including those in the gastrointestinal tract, and have no effect on the central nervous system that may counteract the analgesia. Currently, there are few such medicines, called peripherally acting μ-opioid receptors antagonists (PAMORAs), with peripheral action that seems to be effective and relatively safe [26,27]. They are alvimopan, methylnaltrexone, naloxegol, and naldemedine. This systematic review is focused on the efficacy and safety of the above-mentioned PAMORAs.

## Review

Material and methods

The protocol of this study is registered in the protocol on the International Prospective Register of Systematic Reviews (PROSPERO) with registration number CRD42021256185.

Literature Selection Criteria

We performed an electronic database search in “PubMed,” “Embase,” “CT.gov,” “ICTRP,” and “CINAHL” of the publications that appeared before May 1, 2021. We used a series of logic combinations, word variations and research terms related to opioid-induced constipation and PAMORAs in each database. Published systematic reviews on the same topic were reviewed to identify additional randomized controlled trials. An example of the searching strategy was ("Peripherally acting μ-opioid receptor antagonists" or “Naloxegol” or “Alvimopan” or “Naldemedine” or “Methylnaltrexone” or “Axelopran” or “PAMORA”) AND "opioid-induced constipation" in Title, Abstract, Keyword.

Inclusion and Exclusion Criteria

We included only original studies (RCTs, open-label studies and post-hoc analysis of RCTs) that included cancer and/or non-cancer pain patients as well as patients with advanced illness. We did not limit the results to opioid use for pain relief and included, e.g., patients on methadone for addiction. Other inclusion criteria were trials including adult patients above 18 years of age, on stable opioid doses suffering from OIC. Only publications in the English language, with a full text available, were included in the study. We excluded papers that involved pediatric patients, translational studies on healthy subjects with induced constipation, animal studies, use of PAMORAs for non-opioid constipation, non-completed studies, literature reviews, exploratory studies, intensive care unit (ICU) cohorts.

The focus of this review was to define the efficacy and safety of the studied drugs. Hence, we excluded studies that had no such information. Studies without results, incomplete studies, and duplicate studies were also excluded. We used the Rayyan software tool (Rayyan Systems Inc., Cambridge, MA) [28] for the initial title and abstract screening, followed by a full-text screening in a two-stage process by two independent reviewers. Disagreements were resolved by discussion between authors (MR, GV), followed by consulting an external reviewer, if necessary.

Data Extraction

The two reviewers extracted data independently in a standardized data extraction Excel sheet. Extracted info from each study were study characteristics, year of publication, sample size, age-range and mean, indication and duration for opioids use, type of opioids used, diagnosis of OIC, PAMORA type and dosage, primary outcomes and secondary outcomes, duration of use, effect on OIC, pain scores, side effects, efficacy and use of concomitant laxatives, adverse effect. Any disagreement or queries were resolved by discussion between authors, followed by consulting an external reviewer (MR, GV, AP), if necessary.

Evaluation of the Risk of Bias

Nonrandomized trials were assessed using the Newcastle-Ottawa Scale [29]. Randomized trials were assessed using the Cochrane risk of bias tool [30]. Bias assessments are detailed in Table 2.

**Table 2:** RoB 2 [30]: A revised Cochrane risk-of-bias tool for randomized trials. This table demonstrates the risk of bias across included RCTs. Domain 1: Randomization process Domain 2: Deviation from intended interventions Domain 3: Missing outcome data Domain 4: Measurement of the outcome Domain 5: Selection of the reported results.

Study	Domain 1	Domain 2	Domain 3	Domain 4	Domain 5	Overall risk of bias
Webster et al. [31]	High	Low	Low	Low	Low	High
Tack et al. [32]	Low	Low	Low	Low	Low	Low
Webster et al. [33]	Low	Low	Low	Low	Low	Low
Coyne et al. [34]	Low	Low	Low	Low	Low	Low
Jansen et al. [35]	Low	Low	Low	Low	Low	Low
Webster and Israel [36]	Low	Low	Low	Low	Low	Low
Portenoy et al. [37]	High	Low	Low	Low	Low	High
Thomas et al. [38]	Low	Low	Low	Low	Low	Low
Bull et al. [39]	Low	Low	Low	Low	Low	Low
Iyer et al. [40]	Low	Low	Low	Low	Low	Low
Michna et al. [41]	Low	Low	Low	Low	Low	Low
Rauck et al. [42]	Low	Low	Low	Low	Low	Low
Webster et al. [43]	High	Low	Low	Low	Low	High
Rauck et al. [44]	Low	Low	Low	Low	Low	Low
Yuan et al. [45]	Low	Low	Low	Low	Low	Low
Chamberlain et al. [46]	Low	Low	Low	Low	Low	Low
Lipman et al. [47]	High	Low	Low	Low	Low	High
Nalamachu et al. [48]	Low	Low	Low	Low	Low	Low
Yuan et al. [49]	Low	Low	Low	Low	Low	Low
Wild et al. [50]	Low	Low	Low	Low	Low	Low
Katakami et al. [51]	Low	Low	Low	Low	Low	Low
Katakami et al. [52]	Low	Low	Low	Low	Low	Low
Hale et al. [53]	Low	Low	Low	Low	Low	Low
Webster et al. [54]	Low	Low	Low	Low	Low	Low
Webster et al. [55]	Low	Low	Low	Low	Low	Low
Saito et al. [56]	High	Low	Low	Low	Low	High

Results

**Figure 1:**
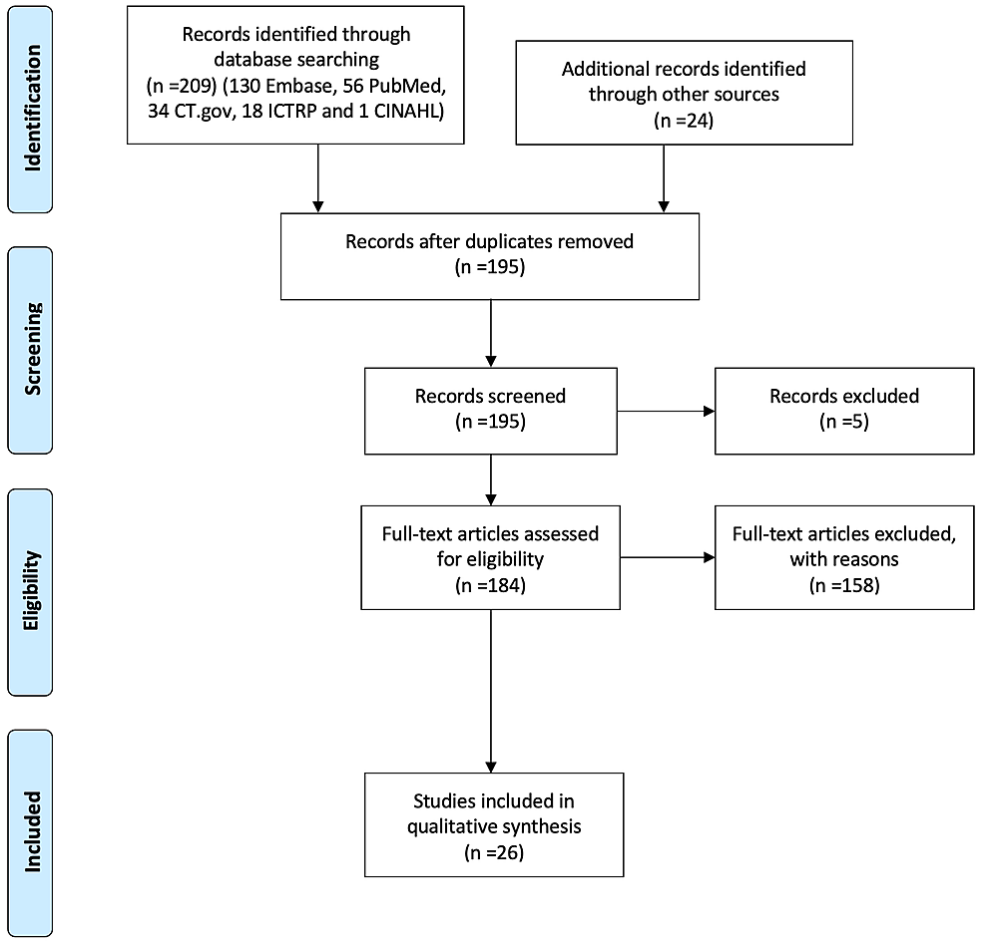
PRISMA flow diagram showing literature search and selection of studies in the analysis.

Our search retrieved 209 trials (130 Embase, 56 PubMed, 34 CT.gov, 18 ICTRP and 1 CINAHL) published from 1996 to 2021; Figure 1). Further screening of articles introduced another 24 papers. After the removal of duplicates, we screened 195 papers. Of them, 5 papers did not have a full text available, and according to the inclusion criteria, only 26 studies were found to be eligible for further analysis. The selected studies represented a total of 11,815 patients. We included one study evaluating alvimopan, 14 studies evaluating methylnaltrexone (10 RCTs and 1 post hoc analysis and 3 open-label studies), 4 studies that evaluated naloxegol (two RCTs [31,32], one open-label study [33] and one post-hoc analysis of RCT [34], 7 studies on naldemedine (all RCTs). Two studies looked at cancer pain, 15 at non-cancer pain, 8 studies include both cancer and non-cancer patients, while 1 did not specify.

Although the number of reported cases is high, the heterogeneity of data did not allow an acceptable meta-analysis. Hence, we decided to limit the study to systematically review the selected papers.

Alvimopan

One study on alvimopan was eligible for this review [35]. This study evaluated a single daily oral dose of 0.5 mg alvimopan versus 0.5 mg twice daily, versus placebo in non-cancer pain patients with OIC for 12 weeks.

Efficacy: A significantly greater proportion of patients in the alvimopan 0.5 mg twice-daily group showed more than three spontaneous bowel movements (SBMs) per week (72% versus 48%, P < 0.001). Moreover, treatment with alvimopan twice daily improved a number of other symptoms compared with placebo and reduced the requirement for rescue laxative use. Alvimopan 0.5 mg once daily produced qualitatively similar but numerically smaller responses than twice-daily treatment. Active treatment did not increase the requirement for opioid medication or in average pain intensity scores [35].

Safety: Over the 12-week treatment period, alvimopan appeared to be well tolerated. There were no statistically significant differences between either dose of alvimopan and placebo over the incidences of treatment-emergent adverse events (TAEs). TAEs affecting the GI system were the most frequently reported (22% in the placebo group, 28% in the once-daily group and 24% in the twice-daily group) [35]. Of note, this single study did not identify the significant severe cardiovascular side effects, which led to approval only for large or small bowel resection surgery with primary anastomosis by FDA (Supplemental Material), but not in Europe.

Methylnaltrexone

Fourteen studies were included; mostly RCTs, one post hoc analysis of RCT [36], and three open-label extended (OLE) studies [37-39]. Six studies included non-cancer pain patients [36,40-44], six studies included both cancer and non-cancer [37,39,45-48], one study included patients with advanced illness [38] and one study did not specify its population [49].

Ten studies used methylnaltrexone as subcutaneous (SC) administration (doses of 0,1 mg/kg, 0.15 mg/kg, 0.3 mg/kg, 1 mg, 5 mg, 12 mg, 20 mg) [37-41,43,46-49]. One study used it intravenously (0.2 mg/kg) [45]. Three studies administered it orally (doses of 150 mg, 300 mg and 450 mg) [36,42,44]. Two of the latter three studies [42,44] utilized the same group of patients but reported either on efficacy or safety of the drug. The duration of the RCTs ranged from 7 hours to 48 weeks and the OLE studies ranged from three weeks to three months. Two studies included patients already on methadone maintenance program [36,49].

Efficacy: All studies showed efficacy in methylnaltrexone as measured by their primary outcome. Most of the studies measured the percentage of patients with rescue free bowel movements within four hours of the first dose. While two studies measured oral cecal transit time [45,49], one study measured constipation symptoms and pain (PAC-SYM) [40] (Table 3).

**Table 3:** Methylnaltrexone efficacy studies. SC: subcutaneous, IV: intravenous.

Dose	Route	Bowel movements	Author
Methylnaltrexone 0.15 mg/kg or 0.3 mg/kg, vs placebo	SC	The median time to bowel movement response was 0.5 hours in the methylnaltrexone group and 2.0 hours in the placebo group (P = 0.013).	Chamberlain et al. [46]
Methylnatrexone 1 mg, 5 mg, 12.5 mg, 20 mg	SC	The median time to laxation was >48 hours for the 1 mg dose group, compared to 1.26 hours for all patients receiving ≥5 mg	Portenoy et al. [37]
Methylnaltrexone (0.15 mg/kg) vs placebo	SC	After the first dose: the median time to bowel movement response was four hours in 48% in the methylnaltrexone group versus 15% in the placebo.	Thomas et al. [38]
Methylnatrexone 8 mg or 12 mg vs placebo once daily	SC	After ≥2 doses: median time to bowel movement response was four hours in 62.9% in the methylnaltrexone group versus 9.6% in the placebo	Bull et al. [39]
Methylnaltrexone 12 mg once daily or placebo	SC	Did not assess bowel movements	Iyer et al. [40]
Methylnatrexone 12 mg once daily, methylnaltrexone 12 mg alternate days vs placebo	SC	58.7% of patients in the methylnaltrexone once-daily group, 45.3% in the alternate-day dosing group, and 38.3% in the placebo group had at least three rescue-free bowel movements per week.	Michna et al. [41]
Methylnatrexone 12 mg once daily	SC	Methylnaltrexone elicited a bowel movement within four hours in 34.1% of the injections throughout the 48-week treatment period.	Webster et al. [43]
Methylnatrexone 0.15 mg/kg as a first dose, adjusted to 0.3 mg/kg or 0.075 mg/kg as needed	SC	Following administration of the first dose through the 15th dose, rescue-free laxation response usually occurred in a median time of 30 minutes or less.	Lipman et al. [47]
Methylnatrexone 0.15 mg/kg, 0.3 mg/kg vs. placebo	SC	More than 50% of patients treated with either methylnaltrexone dose experienced a rescue-free bowel movement within four hours vs. 14.6% of placebo-treated patients. The largest differences vs. placebo were observed for patients taking methylnaltrexone 0.30 mg/kg with a noncancer primary diagnosis and for patients taking methylnaltrexone 0.30 mg/kg maintained on ≥150 mg/day baseline morphine equivalent doses.	Nalamachu et al. [48]
Methylnatrexone 0.1 mg/kg in six subjects, and 0.3 mg/kg in six subjects	SC	Not assessing bowel movements	Yuan et al. [49]
Methylnaltrexone 150, 300 or 450 mg once daily vs. placebo	Oral	Median time to bowel movement response was shorter for patients treated with both oral methylnaltrexone 300 mg and 450 mg. Only the 300 mg dose produced a statistically significant response compared with the placebo	Webster and Israel [36]
Methylnaltrexone 150, 300 or 450 mg once daily vs. placebo	Oral	Median time to bowel movement response was four hours: in 25.4% of patients receiving methylnaltrexone 300 mg; in 23.5% of patients receiving methylnaltrexone 450 mg; in 8% of patients in the placebo group.	Rauck et al. [42]
Methylnatrexone 0.2 mg/kg	IV	Not assessing bowel movements	Yuan et al. [45]

Subcutaneous route: Three studies administered subcutaneous methylnaltrexone or placebo. The first study included patients with advanced illnesses due to cancer and the results showed a higher response in the active treatment group (0.15 mg/kg). Also, the median time to response was statistically significant with 0.5 hours in the methylnaltrexone group (0.15 mg/kg) versus 2 hours in the placebo group [46]. Accordingly, Lipman et al. [47] suggested that the mean response rate for the subcutaneous methylnaltrexone group of 0.15 mg/kg was 45.3% and this remained constant through the three-month open-label study (45.5-57.7%). Similarly, in another study, the mean response rate was 48% for the 0.15 mg/kg SC methylnaltrexone group and only 15% in the placebo group, while this difference between study and placebo groups remained significant after adjustment for baseline opioid dose [38]. Some investigators chose to examine two different subcutaneous doses; 0.15 mg/kg and 0.30 mg/kg of methylnaltrexone. Significantly larger proportion of patients in both study groups found the intervention useful (54.1% in 0.15 mg/kg SC dose, 58.2% in 0.30 mg/kg dose SC, 14,6% in the placebo P<0.001) [48].

Other studies administered a fixed dose of methylnaltrexone rather than by weight. Webster et al. [43] observed a statistically significant increase in mean weekly bowel movements (BM) rate from baseline (mean change = 1.5 BM/wk, P<0.001) in patients on a total SC dose of 12 mg methylnaltrexone once daily during a 48-week period compared to the placebo group. Another study utilized a dose of 12 mg of SC methylnaltrexone that was given either daily or every second day to chronic non-malignant pain patients reporting OIC [41]. Within four hours after the first dose, 34.2% of patients in both methylnaltrexone groups had bowel movements versus 9.9% on placebo [41]. In patients with advanced illness and OIC, subcutaneously administered methylnaltrexone doses between 5 and 20 mg induced a laxation response within four hours significantly more often than a dose of 1 mg (50% versus 10%) [37]. Interestingly, there was no dose-response relationship above 5 mg per day. Bull et al. [39] also tried to assess if a fixed dose of methylnaltrexone (8 mg for under 62 kg and 12 mg for ≥62 kg) would be as efficacious as the doses based on body weight in patients with advanced illness. The percentage of patients achieving rescue-free bowel movements within four hours after ≥2 of the first four doses in the first week was 62.9% and 9.6% for methylnaltrexone and placebo groups, respectively, and was statistically significant.

Yuan et al. [49] trialed two different doses of subcutaneous methylnaltrexone plus morphine, 0.1 mg/kg and 0.3 mg/kg, and found that both dosing regimens reduced the transit times versus the placebo plus morphine. Another finding of this study was that SC methylnaltrexone beyond the advantage of treating OIC, also reduced other unspecified opioid-induced unpleasant subjective symptoms as rated by a 12-item modified opiate adjective checklist.

Iyer et al. [40] studied two dosing regimen schemes for subcutaneous methylnaltrexone and both proved to reduce OIC significantly more than placebo. They were using 12 mg daily or 12 mg every second day, while approximately 60% of the patients were being treated for lower back pain.

Oral route: A significantly greater percentage of patients in oral methylnaltrexone groups 300 mg/d (26.4%, P=0.002) and 450 mg/d (27.4%, P<0.001) achieved mean percentage dosing days that resulted in rescue free bowel movements within four hours of dosing compared with placebo (18.2%). Methylnaltrexone 150 mg also showed improvement compared to placebo (19.9%) but this improvement was not significant [42].

In patients taking concomitant methadone, a greater percentage of patients treated with oral methylnaltrexone 300 mg (33.6%, P<0.01) or 450 mg (38.2%, P<0.001) achieved the same endpoint as the previous study [42] compared to 15.1% in the placebo group. In the same post hoc analysis, the improvements with 150 mg (20.0% of the sample) did not reach statistical significance [36].

Intravenous route: In patients using concomitant methadone, intravenous methylnaltrexone at a dose of 0.365 mg/kg was compared to placebo. The efficacy of the study medication was both proven by statistically significant improved laxation and reduced oral-cecal transit times, as well as higher patient satisfaction in the methylnaltrexone group [45].

Safety: In general, all studies showed that methylnaltrexone is well tolerated in treating OIC in patients with advanced illness and non-cancer pain. Most of the recorded side effects included abdominal pain, flatulence and diarrhea. One study focused just on safety endpoints, such as TAEs [44]. The most commonly reported adverse event (AEs) in the SC methylnaltrexone studies were mild such as abdominal pain [37-40,43,46-48]. This was also the case in patients treated with oral methylnaltrexone [36,42]. In a study involving multiple doses of oral methylnaltrexone, drug-related AEs occurred in higher percentages in patients treated with higher doses [44].

Serious adverse events associated with methylnaltrexone were reported in the form of extrasystoles [41], syncope [37] and non-cardiac chest pain [42]. They all resolved upon discontinuation. In non-cancer patients on long-term opioids, an OLE study 48-week study showed several major adverse coronary events (cardiac arrest, MI, CVA, sudden death) in patients with underlying CV risk factors [43]. Other serious adverse effects were also reported. These were deemed not to be related to the study drug but associated with underlying disease progression, i.e., progression of neoplasm [38,46-48], death [37-39,47], pneumonia [43].

Importantly, pain scores were minimally changed throughout the study compared with baseline in all studies [38-44,46,47]. Additionally, opioid withdrawal symptoms were either none or mild in the methylnaltrexone groups [38,41,43,46,47]. Webster et al. [36] found that hyperhidrosis which was deemed related to opioid withdrawal symptoms; was higher in all the oral methylnaltrexone groups compared to none in the placebo group. In subjects with methadone-induced constipation, no opioid withdrawal symptoms were observed with the use of IV methylnaltrexone [45].

Naloxegol

The included papers were two RCTs [31,32], one OLE study [33], and one post-hoc analysis of RCT [34]. Tack et al. [32] randomized 720 non-cancer patients with symptoms of OIC, that were on a stable dose of opioids (Oral Morphine Equivalent (MEQ)> 30 mg/day) for more than two weeks. The patients were given either oral naloxegol (12.5 mg or 25 mg) or placebo for 12 weeks, while the main conditions treated with opioids were back pain, arthritis, fibromyalgia and joint pain.

Webster et al. [33] reported a multicenter, double-blind RCT which included both cancer and non-cancer patients who were on stable opioid doses for at least two weeks. They randomized 208 patients into three cohorts (oral naloxegol 5 mg, 25 mg and 50 mg) or placebo groups. Patients were stratified into a low opioid group (30-100 daily MEQ) and a high opioid group (30-1000 daily MEQ). All laxatives were discontinued apart from Bisacodyl (an organic compound that is used as a stimulant laxative) if SBM had not occurred in a 72 hours period. The study was conducted over four weeks period [33].

Coyne et al. [34] conducted a post hoc analysis of the assessment of efficacy and safety in patients with non-cancer-related pain and opioid-induced constipation program for naloxegol (KODIAK No4 and No5) which included 1337 patients. The post hoc analysis was a secondary analysis of pooled data from the above studies to examine the relationship between changes from baseline in quality of life (Patient Assessment of Constipation Quality of Life - PAC-QOL), symptoms (Patients Assessment of Constipation-Symptoms - PAC-SYM), stool hardness (Bristol Stool Scale) and rectal straining. The patients had received a daily dose of oral 12.5 or 25 mg of naloxegol or placebo [34].

The fourth is an OLE study, which recruited new patients without prior naloxegol treatment (n=760) or rollover patients from KODIAK No4 and No5 (n = 84). This open-label study assessed the long-term safety and tolerability of naloxegol over the course of 52 weeks. Additionally, non-cancer patients taking 30-1000 MEQ per day for more than four weeks were randomized 2:1 to receive naloxegol 25 mg/day or usual care (investigator-chosen laxative regimen) treatment for OIC [31].

Efficacy: Naloxegol proved to be more effective than placebo in all of the studies (Table 4). In one study, the number of patients having three or more SBM per week was greater in both the naloxegol groups (25 mg, 54.4%; 12.5 mg, 39.2%) compared to placebo (27.2%). SBM was defined as a bowel movement that occurred without the use of rescue laxatives within the previous 24 hours. Also, the median time to first post-dose SBM were 7.6, 19.2 and 41.1 hours for the naloxegol 25 mg, naloxegol 12.5 mg and placebo groups, respectively, indicating significantly greater efficacy of naloxegol 25 mg [32].

**Table 4:** Naloxegol efficacy.

Dose	Bowel movements	Author
Naloxegol 25 mg/d vs usual care	Did not assess spontaneous bowel movements	Webster et al. [31]
Naloxegol 25 mg, 12.5 mg vs placebo	Median time to bowel movement response 7.6 hours for naloxegol 25 mg 19.2 hours for 12.5 mg 41.1 hours for placebo	Tack et al. [32]
Naloxegol 5 mg, 25 mg, 50 mg once daily or placebo	At week 1, the median change from baseline in spontaneous bowel movements per week: In the 5-mg dose group; no statistical difference versus placebo (1.5 vs 1.2) In the 25 mg dose group; a statistically significantly greater change from baseline versus the placebo (2.9 vs 1.0). In the 50-mg dose group, a statistically significantly greater change from baseline versus the placebo (3.3 vs 0.5). At weeks 2-4, the median change from baseline in spontaneous bowel movements per week: For the 50 mg dose group: was statistically significantly greater vs placebo at all time points during weeks 2, 3, and 4. For 25 mg dose group: was statistically significantly greater vs placebo at all time points except week 2	Webster et al. [33]
Naloxegol 25 mg, 12.5 mg vs placebo	Did not assess spontaneous bowel movements	Coyne et al. [34]

Webster et al. [33] reported the median change in SBMs per week from baseline to the end of the first week. This was statistically significant in the 25 mg and 50 mg naloxegol groups; the increase in SBMs was maintained over four weeks for naloxegol 25 mg and 50 mg versus placebo and was statistically significant in both groups. Additionally, the median time to the first laxation was significantly shorter with naloxegol than placebo in the 25-mg cohort (6.6 vs 48.6 hours) and 50-mg cohort (2.9 vs 44.9 hours), but not statistically different in the 5-mg cohort (6.2 vs 28.2 hours; p=0.64).

Tack et al. [32] analyzed the 12-week response rates in the laxative inadequate responders (LIR) group. Patients were classified as LIR if they reported using laxatives for a minimum of four days within two weeks and had continuous stool symptom ratings of moderate, severe or very severe (in response to one or more of the four stool symptom domain questions). SBM response rates and symptoms in the LIR population were significantly higher in both the naloxegol 25 mg and 12.5 mg versus placebo.

Patient Assessment of Constipation - Symptoms (PAC-SYM), Patient Assessment of Constipation - Quality of Life (PAC- QoL), and Short-Form Health Survey (SF-36) scores were also assessed by the studies. Patients treated with 25 mg naloxegol reported lower median total scores on the patient-reported PAC-QoL questionnaire than patients taking a placebo. This group also reported statistically significant improvement in SF-36 scale scores for physical functioning, mental health, social functioning and vitality at various time points during double-blind treatment compared with patients receiving placebo, whereas differences between the 5 mg and 50 mg dose groups and placebo were not significant [33]. In the study of Tack et al. [32], changes from baseline in severity of constipation symptoms as measured by PAC-SYM scores for rectal and stool symptoms were greater for both naloxegol 25 mg and naloxegol 50 mg groups compared with placebo at week 12. PAC-QOL changes from baseline in the satisfaction domain to week 12 were also greater in both naloxegol groups compared to placebo; however, for all other domains (physical discomfort, psychosocial discomfort, worries and concerns) were comparable in both study and placebo groups [32].

Safety and tolerability:* *In these studies, the type, number and frequency of AEs were assessed. Changes from baseline of MEQ dose, Numeric Rating Scale (NRS) pain score, modified Himmelsbach Opioid Withdrawal Score (mHOWS) and Clinical Opiate Withdrawal Scale (COWS) were also assessed [57]. The mHOWS rates yawning, lacrimation, rhinorrhea, perspiration, tremor, mydriasis, piloerection and restlessness on a scale from 0 (none) to 3 (severe). A greater incidence of overall AEs was reported in the naloxegol 25-mg group (63.1%) compared with the naloxegol 12.5-mg (50.6%) or placebo (50.0%) groups resulting in a higher frequency of discontinuation of study drug [32]. The most common AEs reported in the naloxegol group were abdominal pain, diarrhea and nausea [32]. Flatulence, upper abdominal pain and hyperhidrosis were reported more frequently in the naloxegol 25 mg group, versus the naloxegol 12.5-mg or placebo groups [32]. However, Webster et al. [33] reported that there were no major differences in the frequency or type of reported treatment-emergent adverse events (TEAEs) compared with those receiving placebo in patients receiving 5 mg or 25 mg of naloxegol. While most AEs at 5 and 25 mg/day were mild and transient, a difference was noted at the 50 mg cohort where the incidence of TEAEs was higher compared to placebo as was the severity. Accordingly, the most frequently reported TEAEs were GI complaints and included abdominal pain, diarrhea and nausea [33]. A study that compared usual care with naloxegol, stated that the incidence of serious AEs was similar between groups (naloxegol 25 mg, 9.6% and usual care, 11.1%), the treatment-emergent AEs occurring more frequently for naloxegol versus usual care were abdominal pain (17.8% vs. 3.3%), diarrhea (12.9% vs. 5.9%), nausea (9.4% vs. 4.1%), headache (9.0% vs. 4.8%), flatulence (6.9% vs. 1.1%) and upper abdominal pain (5.1% vs. 1.1%). [31].

During the study period, the proportions of patients with increases from baseline in opioid dose, NRS pain score, and mHOWS were similar among treatment groups [32]. Also, Webster et al. [33] stated that there were no significant changes from baseline for mean daily opioid dose for the 25- or 50-mg cohort. Mean NRS scores remained consistent from baseline to week 4 of double-blind treatment for all three cohorts, and no differences vs placebo were observed [33]. Additionally, significant differences in median Clinical Opiate Withdrawal Scale (COWS) total score were observed between placebo and naloxegol in the 5- and 25-mg cohorts. However, a difference was observed in median COWS total score in the 50-mg cohort at day 1 of week 1 compared to placebo (1.0 vs. 0.0; P=0.0069) [33]. In the same study, pain scores and mean daily opioid doses remained stable throughout the study period, with a mean change from baseline of <0.4 on the 0-10 NRS pain scale, for the active treatment group. In patients with noncancer pain and OIC, naloxegol 25 mg was generally safe and well-tolerated over 52 weeks [31].

Naldemedine

Seven RCTs were included, and all of them compared naldemedine to placebo. The age of all subjects was over 18 years, but one study only included a subgroup aged 65-80 years [51]. Most of them studied the use of naldemedine for OIC in non-cancer patients, but two studies included only cancer pain patients [51,52]. Patients with OIC on a stable dose of opioids were included, while the duration of time where patients used opioids ranged from 2 weeks to 4 weeks prior to the study. The studies used oral naldemedine, with most using 0.2 mg once daily; two papers studied three different doses (0.1 mg, 0.2 mg and 0.4 mg) [51,52]. The duration of studies ranged between 2 weeks and 52 weeks, while the OLE study extended to 12 weeks.

Efficacy: All studies reported positive efficacy of one daily dose of oral naldemedine compared to placebo in treating patients with OIC (Table 5). The doses that were evaluated were 0.1 mg, 0.3 mg and 0.4 mg. Hale et al. [53] reported two RCTs (COMPOSE 1 and 2), which randomly assigned patients to receive 0.2 mg oral naldemedine or placebo. The proportion of responders in both trials was significantly higher in naldemedine group than in the placebo group; 47.6% compared to 34.6% (p=0.002) in COMPOSE 1, and 52.5% vs 33.6% (p<0.0001) in COMPOSE 2. A COMPOSE-4 study, utilizing the same oral dose of the study medication, which included only cancer patients, also revealed that the proportion of responders was higher in the naldemedine group (71.1%) vs placebo (34.4%), P<0.001 [55]. In the latter study, a greater change from baseline was observed with naldemedine than with placebo in the frequency of SBMs/week (5.16 v 1.54; p<0.0001) [52].

**Table 5:** Naldemedine efficacy.

Dose	Bowel movements	Author
Naldemedine 0.2 mg vs placebo once daily	Did not assess bowel movements	Wild et al. [50]
Naldemedine 0.1 mg, 0.2 mg, 0.4 mg once daily vs placebo	Change in spontaneous bowel movements frequency (primary endpoint) was higher with all naldemedine doses versus placebo (p<0.05 for all comparisons), as were spontaneous bowel movements responder rates and change in complete spontaneous bowel movements frequency. Change in spontaneous bowel movements frequency without straining was significantly improved with naldemedine 0.2 and 0.4 (but not 0.1) mg versus placebo (at least p<0.05)	Katakami et al. [51]
Naldemedine 0.2 mg once daily vs placebo	The proportion of spontaneous bowel movements responders was significantly greater with naldemedine than with placebo (71.1% vs 34.4). A greater change from baseline was observed with naldemedine than with placebo in the frequency of spontaneous bowel movements/week (5.16 vs 1.54; p<0.0001), spontaneous bowel movements with complete bowel evacuation/week (2.76 vs 0.71; p<0.0001), and spontaneous bowel movements without straining/week.	Katakami et al. [52]
Naldemedine 0.2 mg vs placebo	Not the primary endpoint. Greater increases were observed in the mean frequency of spontaneous bowel movements per week in the naldemedine group than in the placebo group.	Hale et al. [53]
Naldemedine 0.1 mg, 0.2 mg, or 0.4 mg once daily vs placebo	Weekly spontaneous bowel movements frequency was significantly higher with naldemedine 0.2 mg (3.37, p = 0.0014) and 0.4 mg (3.64, p = 0.0003), but not with 0.1 mg (1.98, p = 0.3504), vs placebo (1.42).	Webster et al. [54]
Naldemedine 0.2 mg once daily vs placebo	There was a significant and sustained increase from baseline in the frequency of bowel movements with naldemedine vs placebo throughout the 52-week treatment period.	Webster et al. [55]
Naldemedine 0.2 mg once daily	Did not assess bowel movements	Saito et al. [56]

The beneficial effect of naldemedine on SBM frequency was also supported by a further study [54]. In this, three different doses of oral naldemedine were evaluated (0.1 mg, 0.2 mg and 0.4mg). Although both 0.2 mg and 0.4 mg showed to have a statistically significant effect on weekly SBM versus placebo, the authors concluded that the dose of 0.2 mg is the optimal dose. The 0.1 mg oral dose failed to have a significant change versus placebo [54]. This observation was confirmed by Katakami et al. [51] who trialed the same doses.

Safety: Naldemedine was generally well tolerated in terms of potential adverse effects. One study evaluated the safety and efficacy of naldemedine for up to 12 weeks in a subgroup of patients aged over 65 years from three trials (COMPOSE-1, COMPOSE-2 and COMPOSE-3) [50]. In these studies, the incidence of TEAEs in the naldemedine group (45.9%) was comparable to that in patients receiving placebo (51.6%). The incidence of gastrointestinal system side effects in the naldemedine group (20.2%) was also comparable to that in patients receiving a placebo (16.1%). The incidence of opioid withdrawal in the naldemedine group was 1.1%.

COMPOSE 4 and 5 also assessed the TEAEs (the severity of a TEAE was graded as mild, moderate or severe on the basis of Common Terminology Criteria for Adverse Events, the impact of the TEAE on the daily activities and clinical status of the patient) as well as opioid withdrawal symptoms (assessed with the clinician-administered COWS scoring method) [52]. GI disorders were the most frequently reported TEAE in both COMPOSE 4 and 5 studies, with diarrhea being the most common (COMPOSE-4: naldemedine, 19.6% vs placebo, 7.3%; COMPOSE-5: naldemedine, 18.3% (24 of 131 patients). Vomiting, decreased appetite, pyrexia and abnormal hepatic function test also were attributed to naldemedine use. In COMPOSE-4, TEAEs rate was 44.3% versus the placebo's 26%, P=0.01, while in COMPOSE-5, 80.2% of the patients reported TEAEs. Naldemedine was not associated with signs or symptoms of opioid withdrawal and had no notable impact on opioid-mediated analgesia [52].

Webster et al. [55] studied the long-term safety of naldemedine in the chronic non-cancer population for 52 weeks. TEAEs (naldemedine, 68.4% vs placebo, 72.1%) and TEAEs leading to study discontinuation (6.3% vs 5.8%) were reported for similar proportions of patients. Diarrhea was reported more frequently with naldemedine (11.0%) vs placebo (5.3%). There were no meaningful differences between groups in opioid withdrawal or pain intensity. Saito et al. [56] published COMPOSE 6 and 7 and reported that the most frequent side effects were nasopharyngitis and diarrhea, but were mostly mild or moderate in severity. An increase in pain intensity or opioid withdrawal were not observed.

Discussion

**Figure 2:**
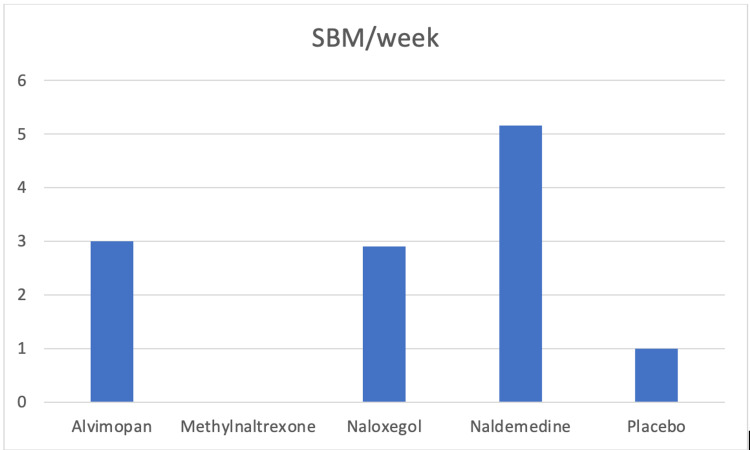
Spontaneous bowel movements per week after some PAMORAs administration. Data deriving from different publications: Almivopan (0.5 mg 2/day), Jansen et al. [35]; Naloxegol (25 mg): Webster et al. [33]; Naldemedine (0.2 mg 1/day), Katakami et al. [51,52]; Methylnaltrexone: SBM are reported just for their speed of appearance, not for the quantity per week. PAMORAs: Peripherally acting μ-opioid receptor antagonists.

**Figure 3:**
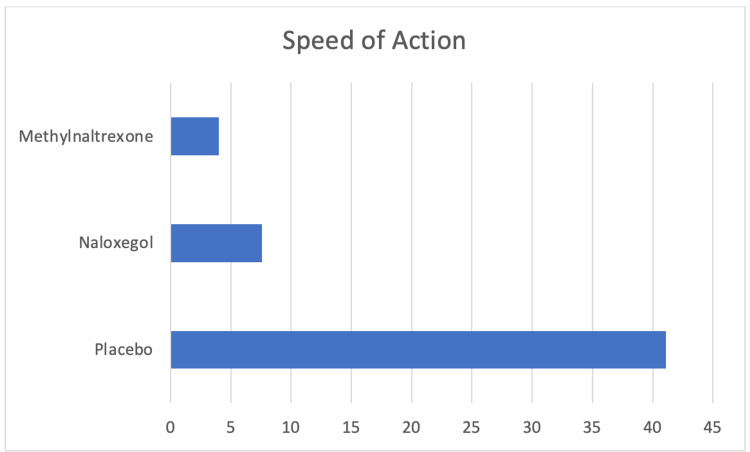
Speed of action (hours after oral administration). Data derived from: methylnaltrexone, Rauck et al. [42]; naloxegol, Tack et al. [32].

All the PAMORAs are efficacious drugs for the treatment of OIC (Table 6). Naldemedine is the PAMORA able to produce the highest number of SBM (Figure 2). It is important to remember that a minimum of three SBM per week is one of the criteria of the Roma IV diagnostic tool for OIC [23]. Methylnaltrexone has the most rapid onset, also when administered orally (Figure 3). It is obvious that alvimopan has a very restricted postoperative use after abdominal surgery and has important adverse effects to consider. Methylnaltrexone, naldemedine and naloxegol have all been available for a prolonged period of time, without serious side effects or complications. Methylnaltrexone can be used for up to four months and has FDA approval for both cancer and non-cancer pain. It is available in both oral and subcutaneous forms. This makes drugs advantageous in some situations such as palliative care; however, the restrictions regarding renal and hepatic impairment, which are common in those patients, impose important limitations. Naldemedine and naloxegol are approved in several countries. Both drugs are not recommended in severe hepatic impairment, but naldemedine has no restrictions in patients with renal impairment making this drug very useful.

**Table 6:** Bowel movements after PAMORAs administration. SC: subcutaneous; PAMORAs: Peripherally acting μ-opioid receptor antagonists.

Dose	Route	Bowel movements	Authors
Almivopan 0.5 mg twice daily or placebo	Oral	≥3 spontaneous bowel movements per week with no laxative use 24 hours before	Jansen et al. [35]
Methylnatrexone 1 mg, 5 mg, 12.5 mg, 20 mg	SC	The median time to laxation was 1.26 hours for all patients receiving ≥5 mg versus>48 hours for the 1 mg dose group	Portenoy et al. [37]
Methylnaltrexone 0.15 mg/kg or placebo	SC	After the first dose: the median time to bowel movement response was four hours in 48% in the methylnaltrexone group versus 15% in the placebo	Thomas et al. [38]
Methylnatrexone 8 mg or 12 mg or placebo once daily	SC	After ≥2 doses: median time to bowel movement response was four hours in 62.9% in the methylnaltrexone group versus 9.6% in the placebo	Bull et al. [39]
Methylnatrexone 12 mg once daily, or 12 mg alternate days or placebo	SC	≥3 spontaneous bowel movements per week with no laxative use 24 hours before: 58.7% of patients in the methylnaltrexone once-daily group 45.3% in the alternate-day dosing group 38.3% in the placebo group	Michna et al. [41]
Methylnatrexone 12 mg once daily	SC	Median time to bowel movement response was four hours in 34.1% of the injections throughout the 48-week treatment period	Webster et al. [43]
Methylnaltrexone 0.15 mg/kg or 0.3 mg/kg or placebo	SC	Median time to bowel movement response 0.5 hours in the methylnaltrexone group versus 2.0 hours in the placebo group	Chamberlain et al. [46]
Methylnatrexone 0.15 mg/kg as a first dose, adjusted to 0.3 mg/kg or 0.075 mg/kg as needed	SC	Median time to bowel movement response 0.5 hours	Lipman et al. [47]
Methylnatrexone 0.15 mg/kg, 0.3 mg/kg or placebo	SC	Median time to bowel movement response was four hours ≥50% in patients receiving either methylnaltrexone dose versus 14.6% of placebo-treated patients. The largest differences vs. placebo were observed for patients taking methylnaltrexone 0.30 mg/kg with a noncancer primary diagnosis and for patients taking methylnaltrexone 0.30 mg/kg maintained on ≥150 mg/day baseline morphine equivalent doses	Nalamachu et al. [48]
Methylnaltrexone 150, 300, 450 mg once daily or placebo	Oral	Median time to bowel movement response was shorter for patients treated with both oral methylnaltrexone 300 mg and 450 mg. Only the 300 mg dose produced a statistically significant response compared with the placebo.	Webster et al. [36]
Methylnaltrexone 150, 300, or 450 mg or placebo once daily	Oral	Median time to bowel movement response was four hours: in 25.4% of patients receiving methylnaltrexone 300 mg. In 23.5% of patients receiving methylnaltrexone 450 mg. In 8% of patients in the placebo group.	Rauck et al. [42]
Naloxegol 25 mg, 12.5 mg or placebo	Oral	Median time to bowel movement response 7.6 hours for naloxegol 25 mg 19.2 hours for 12.5 mg 41.1 hours for placebo	Tack et al. [32]
Naloxegol 5, 25, 50 mg once daily or placebo	Oral	At week 1, the median change from baseline in spontaneous bowel movements per week. In the 5-mg dose group, no statistical difference versus placebo (1.5 vs 1.2). In the 25 mg dose group, a statistically significantly greater change from baseline versus the placebo (2.9 vs 1.0). In the 50 mg dose group, a statistically significantly greater change from baseline versus the placebo (3.3 vs 0.5). At weeks 2-4, the median change from baseline in spontaneous bowel movements per week. For the 50 mg dose group: was statistically significantly greater vs placebo at all time points during weeks 2, 3, and 4. For 25 mg dose group: was statistically significantly greater vs placebo at all time points except week 2.	Webster et al. [33]
Naldemedine 0.1 mg, 0.2 mg, 0.4 mg once daily or placebo	Oral	Spontaneous bowel movements frequency higher (and statistically significant) with all naldemedine doses versus placebo	Katakami et al. [51]
Naldemedine 0.2 mg once daily or placebo	Oral	Spontaneous bowel movements per week were: 5.16 in naldemedine versus 1.54 in the placebo group	Katakami et al. [52]
Naldemedine 0.2 mg or placebo	Oral	Mean frequency of spontaneous bowel movements per week was statistically significant in the naldemedine group: COMPOSE-1: 2.58 vs 1.57 in placebo COMPOSE-2: 2.77 vs 1.62 in the placebo	Hale et al. [53]
Naldemedine 0.1 mg, 0.2 mg, or 0.4 mg once daily or placebo	Oral	Spontaneous bowel movements frequency per week: was significantly higher with naldemedine 0.2 mg (3.37, P = 0.0014) and 0.4 mg (3.64, P = 0.0003) but not with 0.1 mg (1.98, P = 0.3504), vs placebo (1.42)	Webster et al. [54]
Naldemedine 0.2 mg once daily or placebo	Oral	Spontaneous bowel movements frequency per week: a significant and sustained increase from baseline with naldemedine vs placebo throughout the 52-week treatment period	Webster et al. [55]

The only study on alvimopan in OIC showed that an oral dose of 0.5 mg twice daily was effective, and it was in general well tolerated [35]. Alvimopan was approved as a generic drug by FDA, on December 20, 2019, “to accelerate the time to upper and lower gastrointestinal recovery following partial large or small bowel resection surgery with primary anastomosis” [58]. Hence, for the treatment of postoperative ileus after upper and lower gastrointestinal (GI) surgery [59]. The recommended dose is 12 mg administered 30 minutes to 5 hours prior to surgery followed by 12 mg twice daily for up to seven days for a maximum of 15 doses. After the study of Jansen et al. [35], FDA warns of a possible higher risk of myocardial infarctions with long-term use, although a causal relationship with alvimopan has not been established. A short-term treatment only is recommended, making alvimopan an unsuitable agent for patients on long-term opioids [60,61]. This drug could possibly serve as a part of Enhanced Recovery After Surgery protocols (ERAS) [62]. Further studies have to be undertaken in order to establish a definitive conclusion regarding cost-effectiveness, safety and effect on pain scores, as the existing results are inconclusive [63,64].

Methylnaltrexone, in the studies that were included in our results, was actually administered both subcutaneously, intravenously and orally. Table 3 summarizes the findings of each study regarding bowel movements and dosing. Importantly, in studies involving multiple doses of methylnaltrexone, drug-related AEs occurred in higher percentages in patients treated with higher doses (Table 7) and it is worth noting that higher doses did not relate with a better response on some occasions. A metanalysis on methylnaltrexone concluded that 0.15 mg/kg and 0.30 mg/kg doses every other day, as well as a dose of 12 mg/day, were effective [65]. A retrospective analysis including data from the past ten years reports that methylnaltrexone led to a shorter hospital stay and no differences among pain scores or opioid consumption [66]. However, there are some restrictions in dosing of palliative care patients, and also those with renal and hepatic impairment [59]. Moreover, there are warning on potential intestinal perforation due to SC methylnaltrexone administration [67,68].

**Table 7:** Treatment adverse events of PAMORAs. PAMORAS: peripherally acting μ-opioid receptor antagonists, CVA: cerebrovascular accident, MI: myocardial infarction.

Drug/route/dose	Withdrawal effect	Increase in pain	Increased opioid requirements	TAEs	References
Almivopan oral: 0.5-1 mg	Not commented	No	No	Most common: headache, GI system disturbance	[35]
Methylnaltrexone SC: 0.1-0.3 mg/kg or 1-20 mg; IV: 0.2 mg/kg; oral: 150-450 mg	No or mild, e.g., hyperidrosis. In subjects on methadone no withdrawal symptoms [35].	No or minimal change	No or negligible	Most common: abdominal pain, flatulence or diarrhea at a higher dose. Very rare: extrasystoles [41], syncope [37] and non-cardiac chest pain, cardiac arrest, MI, CVA, sudden death (in patients with underlying CV risk factors).	[36-38,41,42, 44-49]
Naloxegol oral: 5-50 mg once daily	None [34] or hyperidrosis more frequent at increased doses [29]	No [33]	No [33]	No difference [33]. Yes - most common: abdominal pain, flatulence or diarrhea, more common at increasing dose [32].	[31-34]
Naldemedine oral: 0.1-0.4 mg	More than placebo, especially over the age of 65 [54]	No [56]	No [56]	Most common: abdominal pain, flatulence or diarrhea at increasing dose. Most commonly reported, nasopharyngitis and diarrhea [61]. Very rare: CVA (not connected to the used medicine) [58].	[50-56]

Naloxegol 12.5 mg, 25 mg and 50 mg orally proved to be more effective than placebo in all the studies, but not at the dose of 5 mg. The results among the effective doses were comparable as well. In general, a greater incidence of overall AEs such as diarrhea, nausea, headache, flatulence, upper abdominal pain, and hyperhidrosis was reported in higher doses by some studies, whereas other studies did not find any differences among 5 mg and 25 mg, but only between 25 mg and 50mg, where the incidence and severity of AEs were significantly higher in the group receiving the higher dose [33]. This is also supported by a previous review where it is stated that the recommended dosage of naloxegol is 25 mg once daily in the morning before food intake, while under certain circumstances, the recommended dosage is 12.5 mg/day (e.g., tolerability issues/ drug interactions, or as a starting dosage in patients with moderate, severe or end-stage renal impairment) [69]. Finally, in the summary of product characteristics published by the European Medicines Agency (EMA) for naloxegol, the recommended dose is 25 mg once daily [70]. When naloxegol therapy is initiated, it is recommended that all currently used maintenance laxative therapy should be halted, until the clinical effect of naloxegol is determined. No dose adjustment is recommended based on age, but the starting dose is 12.5 mg in severe renal insufficiency. Use in patients with severe hepatic impairment is not recommended. If side effects impacting tolerability occur, naloxegol should be discontinued or the dose to be decreased [70].

Effects of oral naldemedine 0.1 mg were not significantly different compared to placebo. Results obtained with 0.2 mg and 0.4 mg doses were significantly better in all the studies using these dosages. Additionally, a metanalysis showed that the dose of naldemedine 0.2 mg daily provided a significant reduction of symptoms in patients with OIC and was generally well tolerated [71]. Another meta-analysis concluded that there was a significant difference between the naldemedine 0.1 mg and 0.2 mg group for treatment efficacy, but there were no differences between 0.2 mg and 0.4 mg [72]. Regarding the adverse effects, some studies showed that there is no difference between the studied drug and placebo. Others showed a higher frequency of AEs and more discontinuation in the treatment group. Also, they showed that the incidence of serious adverse effects (AEs) was higher with naldemedine than with placebo, especially in the cancer patient subgroup, but they were mild to moderate and well-tolerated during treatment [72]. A study that included two RCTs confirmed that naldemedine, at a dose of 0.2 mg, benefits patients with OIC and cancer irrespective of baseline characteristics [73]. Also, this dose did not appear to affect analgesia or produce withdrawal symptoms. Pharmacokinetic assessments indicate that dose adjustments for naldemedine are not necessary for subjects with any degree of renal impairment [74,75] or for subjects with mild or moderate hepatic impairment [74], which makes the drug a very useful agent for OIC in a relatively large group of patients treated with opioids.

Finally, naloxegol is not recommended when used with CYP3A4 inhibitors and inducers, while naldemedine can be used with the indications to monitor/decrease the doses if needed [76]. Considering what is reported above, the choice of the right PAMORA depends on many clinical components, but there are clear rules that should be followed [77].

Limitations

This study has several limitations. The most important is that a credible meta-analysis to compare the results obtained with the different PAMORAs cannot be done due to the heterogeneity between studies. The available data for the four PAMORAs are very different and the comparative analysis of some of them is very difficult. As an example, the number of SBM per week, reported for alvimopan, naloxegol and naldemedine, has not been specifically evaluated for methylnaltrexone. For this last drug, there are much data on the rapidity of response to the injection [44]. Further, there are no head-to-head studies, so direct comparisons are not possible. Also, we just examined the data on “efficacy” and “safety.” Other aspects would have been interesting as well, e.g., the survival of patients affected by OIC treated or not-treated with PAMORAs like it has been reported for methylnaltrexone in advanced cancer patients [78]. Moreover, between the adverse events, we did not study the abdominal pain evoked by PAMORAs and its relationship with laxation, which has resulted very frequently in some groups of patients [79,80].

## Conclusions

OIC is an important side effect of acute and chronic opioid usage. PAMORAs seem to be effective and relatively safe. Higher doses seem to have more sides effects and also are not always connected with better outcomes. Also, there are specific indications, such as that for alvimopan in postoperative ileus. The different formulations available provide a large armamentarium to the clinicians. For example, palliative care patients and patients with advanced disease could benefit from subcutaneous administration, possibly with methylnaltrexone. While non-cancer patients chronically treated with opioids would be better treated with oral drugs, like naldemedine.

## Appendices

**Table 8:** Supplemental material These are the indications of the Food and Drugs Administration (FDA) of the USA for the four PAMORAs [59].

PAMORA/Commercial name	Dose	Route	FDA (approval/indication)	Renal/hepatic impairment dose adjustment	Restrictions	Source
Alvimopan Entereg (initial U.S approval 2008)	Caps 12 mg	Oral	Accelerate the time to upper and lower gastrointestinal recovery following partial large or small bowel resection surgery with primary anastomosis	Mild-to-moderate hepatic impairment: do not require dosage adjustment/monitor for adverse effects not recommended for patients with severe hepatic impairment. Renal impairment: Alvimopan has not been studied in patients with end-stage renal disease/not recommended for use in these patients. Dosage adjustment is not required in patients with mild to severe renal impairment but they should be monitored for adverse effects.	Entereg is available only for short-term (15 doses) use in hospitalized patients. Contraindications: -.Therapeutic doses of opioids for more than seven consecutive days prior to Entereg	https://www.accessdata.fda.gov/drugsatfda_docs/label/2008/021775lbl.pdf
Methylnaltrexone Relistor (initial U.S approval 2008)	Tablets: 150 mg. Injection: 8 mg or 12 mg. Once-daily.	Oral/SC	OIC non-cancer/ cancer/ palliative patients with chronic pain single SC dose of 12 mg once daily if required as 4–7 doses weekly. In palliative care the SC doses recommended are: For Adult (body-weight up to 38 kg): 150 micrograms/kg once daily on alternate days for a maximum duration of treatment 4 months, two consecutive doses may be given 24 hours apart if no response to treatment on a preceding day. For Adult (body-weight 38–61 kg): 8 mg once daily on alternate days for a maximum duration of treatment of 4 months, two consecutive doses may be given 24 hours apart if no response to treatment on the preceding day. For Adult (body-weight 62–114 kg): 12 mg once daily on alternate days for a maximum duration of treatment of 4 months, two consecutive doses may be given 24 hours apart if no response to treatment on the preceding day. For adults (body-weight 115 kg and above): 150 mg/kg once daily on alternate days for a maximum duration of treatment 4 months, two consecutive doses may be given 24 hours apart if no response to treatment on the preceding day.	Dosage reduction in severe renal impairment moderate or severe hepatic impairment	Do not use in known or suspected mechanical gastrointestinal obstruction and at increased risk of recurrent obstruction. Use beyond four months has not been studied in the advanced illness population.	FDA (https://www.accessdata.fda.gov/drugsatfda_docs/label/2017/021964s018,208271s002lbl.pdf)
Naldemendine Symproic. Approval date: March 23, 2017.	0.2 mg OD	Oral	OIC in adult patients with chronic non-cancer pain	Avoid severe hepatic impairment	Do not use in patients with a known or suspected gastrointestinal obstruction or at increased risk of recurrent obstruction. Strong CYP3A inducers (e.g., rifampin): decreased naldemedine concentrations; avoid concomitant use. Moderate (e.g., fluconazole) and strong (e.g., itraconazole) CYP3A4 inhibitors: increased naldemedine concentrations; monitor for adverse reactions	https://www.accessdata.fda.gov/drugsatfda_docs/label/2008/021775lbl.pdf
Naloxegol Movantik. Initial US Approval: 2014.	Tablets: 12.5 mg and 25 mg.	Oral	OIC in adult patients with chronic non-cancer pain	Avoid severe hepatic impairment in renal impairment start with a lower dose in moderate and severe renal impairment - no dose adjustment needed for mild.	Do not use in patients with known or suspected gastrointestinal obstruction and at increased risk of recurrent obstruction concomitant use with: strong CYP3A4 inhibitors (e.g., clarithromycin, ketoconazole): contraindicated strong CYP3A4 inducers (e.g., rifampin): contraindicated moderate CYP3A4 inducers (e.g., diltiazem, erythromycin, verapamil): reduce dosage to 12.5 mg	https://www.accessdata.fda.gov/drugsatfda_docs/label/2014/204760s000lbl.pdf
